# Tricritical wings and modulated magnetic phases in LaCrGe_3_ under pressure

**DOI:** 10.1038/s41467-017-00699-x

**Published:** 2017-09-15

**Authors:** Udhara S. Kaluarachchi, Sergey L. Bud’ko, Paul C. Canfield, Valentin Taufour

**Affiliations:** 1 The Ames Laboratory, US Department of Energy, Iowa State University, Ames, IA 50011 USA; 2 Department of Physics and Astronomy, Iowa State University, Ames, IA 50011 USA; 3 Present Address: Department of Physics, University of California, Davis, CA 95616 USA

## Abstract

Experimental and theoretical investigations on itinerant ferromagnetic systems under pressure have shown that ferromagnetic quantum criticality is avoided either by a change of the transition order, becoming of the first order at a tricritical point, or by the appearance of modulated magnetic phases. In the first case, the application of a magnetic field reveals a wing-structure phase diagram as seen in itinerant ferromagnets such as ZrZn_2_ and UGe_2_. In the second case, no tricritical wings have been observed so far. Here, we report on the discovery of wing-structure as well as the appearance of modulated magnetic phases in the temperature-pressure-magnetic field phase diagram of LaCrGe_3_. Our investigation of LaCrGe_3_ reveals a double-wing structure indicating strong similarities with ZrZn_2_ and UGe_2_. But, unlike these simpler systems, LaCrGe_3_ also shows modulated magnetic phases similar to CeRuPO. This finding provides an example of an additional possibility for the phase diagram of metallic quantum ferromagnets.

## Abstract (web summary)

The study of phase transitions in quantum ferromagnets has shown that the approach to a continuous quantum ferromagnetic transition is typically interrupted by either a tricritical point or a new magnetic phase. Here the authors show that LaCrGe_3_ exhibits both these features in its phase diagram.

## Introduction

Suppressing a second-order, magnetic phase transition to zero temperature with a tuning parameter (pressure, chemical substitutions, magnetic field) has been a very fruitful way to discover many fascinating phenomena in condensed matter physics. In the region near the putative quantum critical point (QCP), superconductivity has been observed in antiferromagnetic^1^ as well as ferromagnetic (FM) systems^2–4^. One peculiarity of the clean FM systems studied so far is that the nature of the paramagnetic-ferromagnetic (PM-FM) phase transition always changes before being suppressed to zero temperature^5^: in most cases, the transition becomes of the first order^6–11^. Recently, another possibility, where a modulated magnetic phase (AFM_*Q*_) appears (spin-density wave, antiferromagnetic order), has been observed in CeRuPO^12, 13^, MnP^14, 15^, and LaCrGe_3_^16^.

**Fig. 1:**
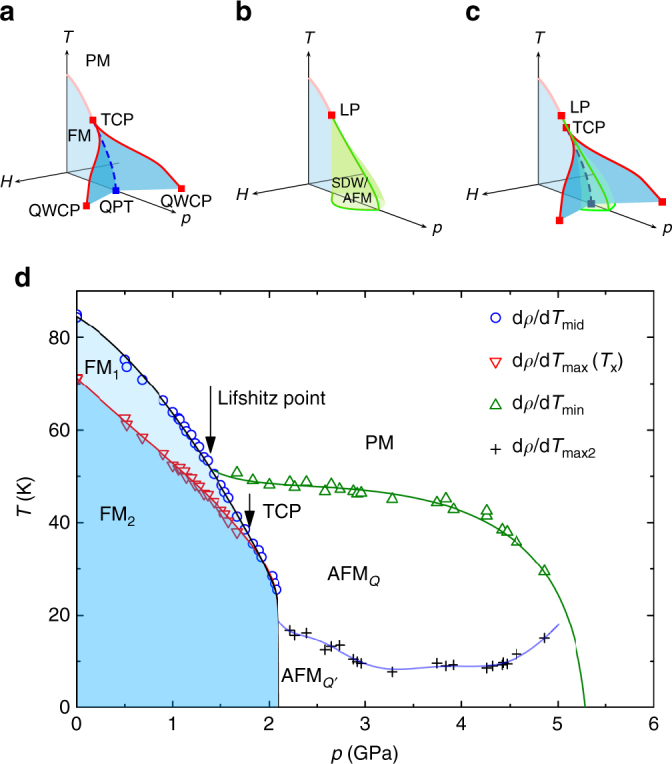
Temperature–pressure phase diagram. **a** Schematic *T*-*p*-*H* phase diagram of a quantum ferromagnet: the paramagnetic-ferromagnetic (PM-FM) transition becomes of the first order at a tricritical point (TCP) after which there is a quantum phase transition (QPT) at 0 K. Tricritical wings emerge from the TCP under magnetic field and terminate at quantum wing critical points (QWCP). **b** Schematic *T*-*p*-*H* phase diagram of a quantum ferromagnet when a modulated magnetic phase (SDW/AFM) emerges from the Lifshitz point (LP). **c** New possible schematic *T*-*p*-*H* phase diagram for which tricritical wings as well as a new magnetic phase are observed. **d** *T*-*p* phase diagram of LaCrGe_3_ from electrical resistivity measurements^16^ showing two FM regions (FM1 and FM2) separated by a crossover. The *solid lines* are guides to the eye

Several theories have been developed to explain those possibilities^17–26^. When a FM transition becomes of the first order at a tricritical point (TCP) in the temperature *T* pressure *p* plane, the application of a magnetic field *H* along the magnetization axis reveals a wing structure phase diagram in the *T*-*p*-*H* space^20, 27^. This is seen in UGe_2_^28, 29^ and ZrZn_2_^30^ and is schematically represented in Fig. 1a. This phase diagram shows the possibility of a different kind of quantum criticality at the quantum wing critical point (QWCP). In contrast with the conventional QCP, symmetry is already broken by the magnetic field at a QWCP. In the more recently considered case where the transition changes to a AFM_*Q*_ phase, no wing structure phase diagram has been reported, but it is found that the AFM_*Q*_ is suppressed by moderate magnetic field^12, 13^. This second possible *T*-*p*-*H* phase diagram has been schematically presented in a recent review^5^ and reproduced in Fig. 1b.

Here, we report electrical resistivity measurements on LaCrGe_3_ under pressure and magnetic field. We determine the *T*-*p*-*H* phase diagram and find that it corresponds to a third possibility where tricritical wings emerge in addition to the AFM_*Q*_ phase. This type of phase diagram is illustrated in Fig. 1c: it includes both the tricritical wings and the AFM_*Q*_ phase. In addition, the phase diagram of LaCrGe_3_ shows a double wing structure similar to what is observed in the itinerant ferromagnets UGe_2_^31^ and ZrZn_2_^32^, but with the additional AFM_*Q*_ phase. LaCrGe_3_ is the first example showing such a phase diagram.

## Results

### *T*-*p* phase diagram

Recently, we reported on the *T*-*p* phase diagram of LaCrGe_3_^16^, which is reproduced in Fig. 1d. At ambient pressure, LaCrGe_3_ orders ferromagnetically at *TC* = 86 K. Under applied pressure, *TC* decreases and disappears at 2.1 GPa. Near 1.3 GPa, there is a Lifshitz point^33^ at which a new transition line appears. The transition corresponds to the appearance of a modulated magnetic phase (AFM_*Q*_) and can be tracked up to 5.2 GPa. Muon-spin rotation (μSR) measurements show that the AFM_*Q*_ phase has a similar magnetic moment as the FM phase but without net macroscopic magnetization^16^. In addition, band structure calculations suggest that the AFM_*Q*_ phase is characterized by a small wave-vector *Q* and that several small *Q* phases are nearly degenerate. Below the PM-AFM_*Q*_ transition line, several anomalies marked as gray cross in Fig. 1d can be detected in *ρ*(*T*)^16^. These other anomalies within the AFM_*Q*_ phase are compatible with the near degeneracy of different *Q*-states (shown as AFM_*Q*_ and AFM_*Q*′_) with temperature and pressure driven transitions between states with differing wavevectors.

In this article, we determine the three dimensional *T*-*p*-*H* phase diagram of LaCrGe_3_ by measuring the electrical resistivity of single crystals of LaCrGe_3_ under pressure and magnetic field.

### FM1 and FM2 phases

Whereas most of the features in Fig. 1d were well understood in ref. ^16^, we also indicate the pressure dependence of *T*_*x*_ (d*ρ*/d*T*_max_) at which a broad maximum is observed in d*ρ/*d*T* below *TC* and shown as orange triangles in Fig. 1d. At ambient pressure, *T*_*x*_ ≈ 71 K. No corresponding anomaly can be observed in magnetization^16^, internal field^16^ or specific heat^34^. Under applied pressure, *T*_*x*_ decreases and cannot be distinguished from *TC* (d*ρ*/d*T*_mid_) above 1.6 GPa. As will be shown, application of magnetic field allows for a much clearer appreciation and understanding of this feature.

**Fig. 2:**
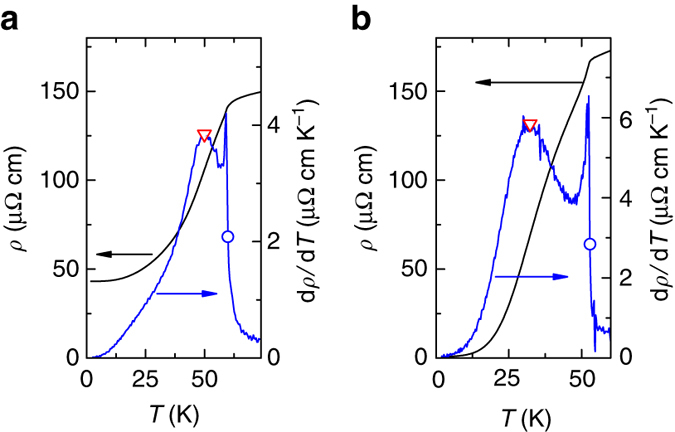
Comparison of *ρ*(*T*) and its d*ρ*(*T*)/d*T* between LaCrGe_3_ and UGe_2_. Temperature dependence of the resistivity (*black line*, *left axis*) and its derivative (*blue line*, *right axis*) of **a** LaCrGe_3_ at 1.14 GPa and **b** UGe_2_ at 0 GPa from ref. ^28^. The crossover between the two ferromagnetic phases (FM1 and FM2) is inferred from the maximum in d*ρ*/d*T* (*T*_*x*_) and marked by a *red triangle*, whereas the paramagnetic-ferromagnetic transition is inferred from the middle point of the sharp increase in d*ρ*/d*T* (*TC*) and indicated by a *blue circle*

Figure 2a shows the anomalies at *T*_*x*_ and *T*_C_ observed in the electrical resistivity and its temperature derivative at 1.14 GPa. For comparison, Fig. 2b shows ambient pressure data for UGe_2_^28^ where a similar anomaly at *T*_*x*_ can be observed. In UGe_2_, this anomaly was studied intensively^35–37^. It corresponds to a crossover between two ferromagnetic phases FM1 and FM2 with different values of the saturated magnetic moment^35, 36^. Under pressure, there is a critical point at which the crossover becomes a first-order transition, which eventually vanishes where a maximum in superconducting-transition temperature is observed^2^. In the case of LaCrGe_3_, we cannot locate where the crossover becomes a first order transition, since the anomaly merges with the Curie temperature anomaly near 1.6 GPa, very close to the TCP. However, as we will show below, the two transitions can be separated again with applied magnetic field above 2.1 GPa. This is similar to what is observed in UGe_2_ where the PM-FM1 and FM1-FM2 transition lines separate more and more as the pressure and the magnetic field are increased. Because of such similarities with UGe_2_, we label the two phases FM1 and FM2 and assume that the anomaly at *T*_*x*_ corresponds to a FM1-FM2 crossover. A similar crossover was also observed in ZrZn_2_^32^. In refs ^18, 25^, a Stoner model with two peaks in the density of states near the Fermi level was proposed to account for the two phases FM1 and FM2, reinforcing the idea of the itinerant nature of the magnetism in LaCrGe_3_.

### Field-dependent resistivity measurement under pressure

**Fig. 3:**
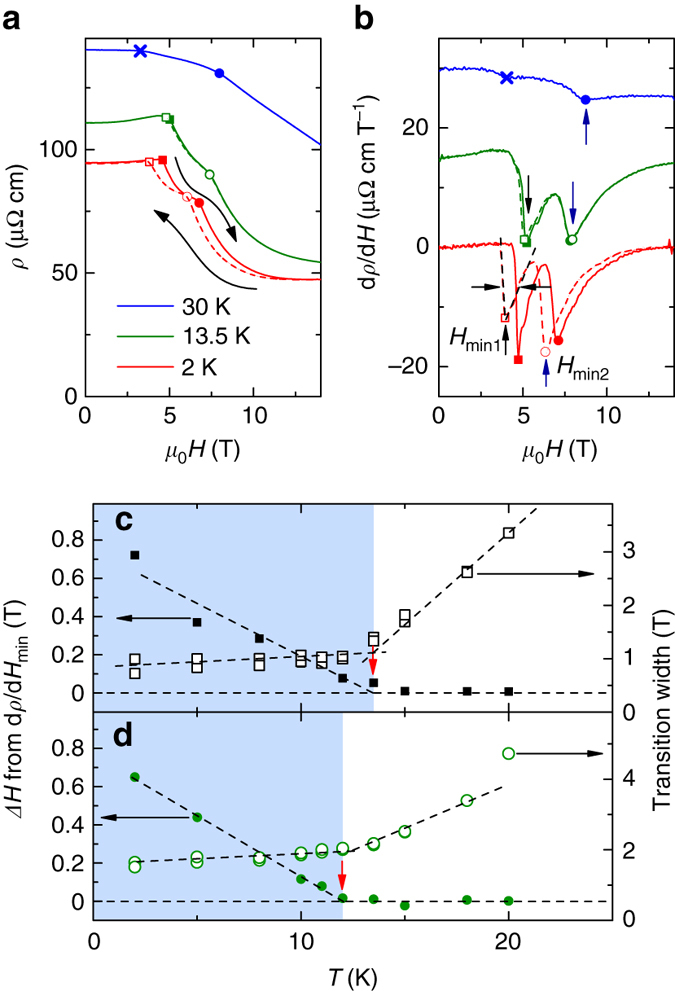
Determination of wing critical point at 2.39 GPa. **a** Field dependence of the electrical resistivity at 2, 13.5, and 30 K at 2.39 GPa with applied field along the *c*-axis. *Continuous* and *dashed lines* represent the field increasing and decreasing, respectively. When the *continuous* and *dashed lines* do not overlap, there is indication for hysteresis. **b** Corresponding field derivatives (d*ρ*/d*H*). The curves are shifted by 15 μΩ cm T^−1^ for clarity. *Vertical arrows* represent the minima. The transition width is determined by the full width at half minimum as represented by *horizontal arrows*. *Solid* and *open symbols* in **a**, **b** represent the transition fields for field increasing and decreasing (*squares* for AFM_*Q*_-FM1 and *circles* for FM1-FM2). The *blue cross symbols* in **a**, **b** represent the AFM_*Q*_-PM transition at this pressure. The temperature dependence of the hysteresis width of *H*_min1_ and *H*_min2_ (*solid symbols*) are shown in **c**, **d** (*left axes*). The hysteresis width gradually decreases with increasing temperature and disappears at *Tw* corresponding to the wing critical point (*vertical red arrows*). The *right axes* show the temperature dependence of the transition widths (*open symbols*). *Dashed lines* are guides to the eye. The width is small for the first-order transition and becomes broad in the crossover region. The *blue-color* *shaded area* represents the first order transition region, whereas the *white color* *area* represents the crossover region. These allow for the determination of the wing critical point of the FM1 transition at 13.5 ± 1.5 K, 2.39 GPa, and 5.1 T and the one for the FM2 transition at 12 ± 1 K, 2.39 GPa and 7.7 T

In zero field, for applied pressures above 2.1 GPa, both FM1 and FM2 phases are suppressed. Upon applying a magnetic field along the *c*-axis, two sharp drops of the electrical resistivity can be observed (Fig. 3a) with two corresponding minima in the field derivatives (Fig. 3b). At 2 K, clear hysteresis of Δ*H* ~ 0.7 T can be observed for both anomalies indicating the first order nature of the transitions. The emergence of field-induced first-order transitions starting from 2.1 GPa and moving to higher field as the pressure is increased (Supplementary Note 1) is characteristic of the FM quantum phase transition: when the PM-FM transition becomes of the first order, a magnetic field applied along the magnetization axis can induce the transition resulting in a wing structure phase diagram such as the one illustrated in Fig. 1a. In the case of LaCrGe_3_, evidence for a first order transition was already pointed out because of the very steep pressure dependence of *T*_C_ near 2.1 GPa and the abrupt doubling of the residual (*T* = 2 K) electrical resistivity^16^. In UGe_2_ or ZrZn_2_, the successive metamagnetic transitions correspond to the PM-FM1 and FM1-FM2 transitions. In LaCrGe_3_, at 2 K, due to the presence of the AFM_*Q*′_ phase at zero field, the transitions correspond to AFM_*Q*′_-FM1 and FM1-FM2.

### Determination of the wing structure phase diagram

As the temperature is increased, the hysteresis decreases for both transitions, as can be seen in Fig. 3c, d and disappears at a wing critical point (WCP). Also, the transition width is small and weakly temperature dependent below the WCP and it broadens when entering in the crossover regime. Similar behavior has been observed in UGe_2_^29^. At 2.39 GPa for example, we locate the WCP of the first-order FM1 transition around 13.5 K and the one of the first-order FM2 transition around 12 K. At this temperature and pressure, the transitions occur at 5.1 and 7.7 T, respectively. This allows for the tracking of the wing boundaries in the *T*-*p*-*H* space up to our field limit of 14 T. At low field, near the TCP, the wing boundaries are more conveniently determined as the location of the largest peak in d*ρ*/d*T* (Supplementary Note 2).

**Fig. 4:**
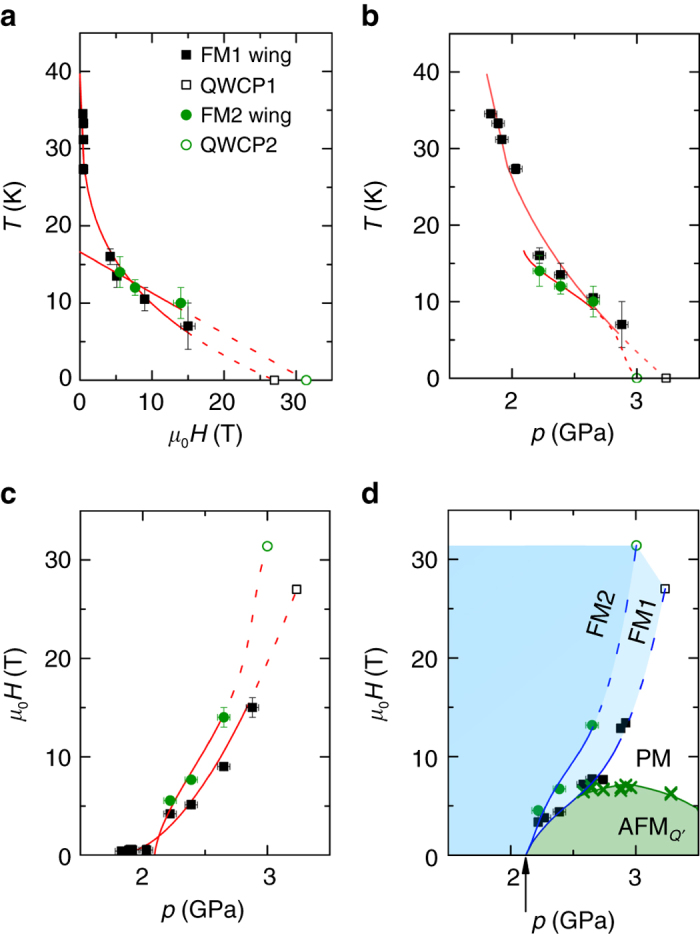
Shape of the tricritical wing lines (*T*_w_, *p*_w_, *H*_w_). Projection of the wings in **a** *T*-*H*, **b** *T*-*p* and **c** *H*-*p* planes. *Black solid squares* and *green solid circles* represent the FM1-wing and FM2-wing, respectively. *Red lines* (represented in the *T*-*p*-*H* space in Fig. 5) are guides to the eyes and *open symbols* represent the extrapolated quantum wing critical point (QWCP). **d** *H*-*p* phase diagram at 2 K. The *arrow* represents the pressure *p*_*c*_ = 2.1 GPa. The *green color* *cross symbols* represent the AFM_*Q*′_ phase boundary. *Dark blue*, *light blue*, and *green color* *shaded areas* represent the FM2, FM1, and AFM_*Q*′_ phases, respectively. The *error bars* in pressure for **b**–**d** are determined by the superconducting transition width of the Pb manometer. For **a**–**d**, the *error bars* in temperature and magnetic field are determined as half the data spacing

The projections of the wings lines *T*_w_(*p*, *H*) in the *T*-*H*, *T*-*p*, and *H*-*p* planes are shown in Fig. 4a–c, respectively. The metamagnetic transitions to FM1 and FM2 start from 2.1 GPa and separate in the high field region as the pressure is further increased. For the FM1 wing, the slope d*T*_w_/d*H*_w_ is very steep near *H* = 0 (Fig. 4a), whereas d*H*_w_/d*p*_w_ is very small (Fig. 4c). This is in agreement with a recent theoretical analysis based on the Landau expansion of the free energy, which shows that d*T*_w_/d*H*_w_ and d*p*_w_/d*H*_w_ are infinite at the TCP^27^. This fact was overlooked in the previous experimental determinations of the wing structure phase diagram in UGe_2_^28, 29^ and ZrZn_2_^30^, but appears very clearly in the case of LaCrGe_3_. In the low field region, there are no data for the FM2 wing since the transition is not well separated from the FM1 wing, but there is no evidence for an infinite slope near *H* = 0. The wing lines can be extrapolated to QWCPs at 0 K in high magnetic fields of the order of ~30 T (Fig. 4a) and pressures around ~3 GPa (Fig. 4b).

Figure 4d shows the *H*-*p* phase diagram at low temperature (*T* = 2 K). The magnetic field at the transition to the FM1 phase increases rapidly, whereas the field suppressing the AFM_*Q*′_ phase does not exceed 7 T. Above 2.5 GPa, the AFM_*Q*′_ and FM1 phases are separated by a region corresponding to the polarized PM phase. We note that a similar diagram where the wings extend beyond the AFM phase was recently obtained theoretically in the case of strong quantum fluctuations effects^38^. The similarity of the *H*-*p* phase diagram at 2 K (Fig. 4d) and the projection of the wings in the *H*-*p* plane (Fig. 4c) reveals the near vertical nature of the wings.

### *T-p-H* phase diagram of LaCrGe_3_

**Fig. 5:**
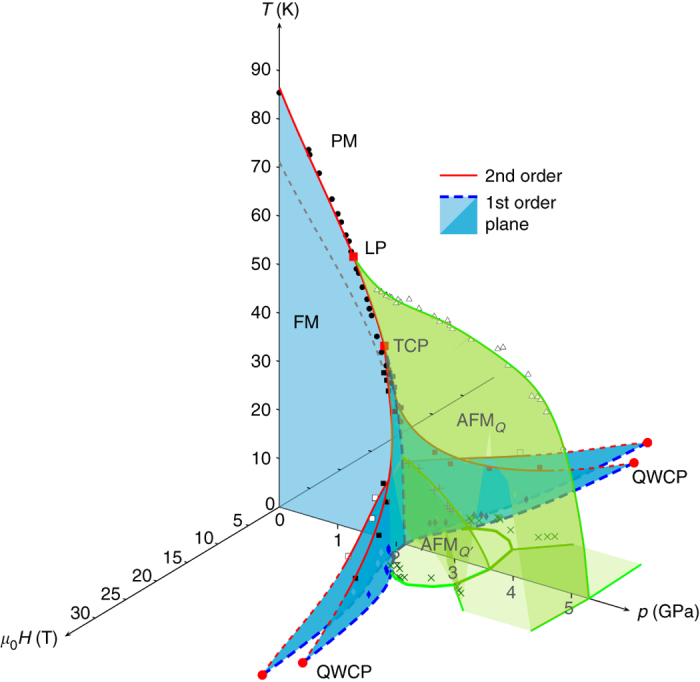
*T*-*p*-*H* phase diagram of LaCrGe_3_. *Red solid lines* are the second order phase transition and *blue color* *planes* are planes of first order transitions. *Green color* *areas* represent the AFM_*Q*_ and AFM_*Q*′_ phases. For clarity, only data points at 2 K and along the transitions lines are shown. *Black circles* indicate the paramagnetic (PM)-ferromagnetic (FM) transition. *Black squares* indicate the wing critical line for FM1 and *empty squares* indicate the wing critical line for FM2. *Blue diamonds* indicate the first order transition to the FM1 state at 2 K and *empty diamonds*, the first order transition to FM2 at 2 K. *Empty triangles* indicate the PM-modulated magnetic phase (AFM_*Q*_) and “+” indicate the AFM_*Q*′_ boundary. “*×*” indicate the AFM_*Q*_ or AFM_*Q*′_ boundary at 2 K. The full set of data points can be found in Supplementary Note 3. For clarity, the *green surfaces* representing the boundaries for the AFM_*Q*_ or AFM_*Q*′_ phases are shown only in the region of positive magnetic field

The resulting three-dimensional *T*-*p*-*H* phase diagram of LaCrGe_3_ is shown in Fig. 5, which summarizes our results (Several of the constituent *T*-*H* phase diagrams, at various pressures, are given in Supplementary Fig. 3). The double wing structure is observed in addition to the AFM_*Q*_ phase. This is the first time that such a phase diagram is reported. Other materials suggested that there is either a wing structure without any additional magnetic phase^28–30^, or a magnetic phase without a wing structure^12, 13^. The present study illustrates a third possibility where all such features are observed. The phase diagram of LaCrGe_3_ and the existence of wings clearly establish that the quantum phase transition from FM to AFM_*Q*′_ is of the first order. It is plausible that the reason is the same as for the FM to PM transition, but no theory is available for this case, as pointed out in a recent review^5^. Another interesting aspect is the existence of the two metamagnetic transitions (to FM1 and FM2) which suggests that this might be a generic feature of itinerant ferromagnetism. Indeed, it is observed in ZrZn_2_, UGe_2_, and LaCrGe_3_, although these are very different materials with different electronic orbitals giving rise the the magnetic states. We note that a wing structure has also been determined in the PM compounds UCoAl^39–41^ and Sr_3_Ru_2_O_7_^42^, implying that a FM state probably exists at negative pressures in these materials. Strikingly, two anomalies could be detected upon crossing the wings in UCoAl (two kinks of a plateau in electrical resistivity^39^, two peaks in the ac susceptibility^41^), as well as in Sr_3_Ru_2_O_7_ (two peaks in the ac susceptibility^42^). These double features could also correspond to a double wing structure.

### Conclusions

To conclude, the *T*-*p*-*H* phase diagram of LaCrGe_3_ provides a distinct example of possible outcomes of FM quantum criticality. At zero field, quantum criticality is avoided by the appearance of a new modulated magnetic phase, but the application of magnetic field shows the existence of a wing structure phase diagram leading towards QWCP at high field. These experimental findings reveal insights into the possible phase-diagrams of FM systems. The emergence of the wings reveals a theoretically predicted tangent slope^27^ near the TCP, a fact that was overlooked in previous experimental determination of phase diagrams of other compounds because of the lack of data density in that region. In addition, the double nature of the wings appears to be a generic feature of itinerant ferromagnetism, as it is observed in several, a priori, unrelated materials. This result deserves further theoretical investigations and unification.

## Methods

### Sample preparation

Single crystals of LaCrGe_3_ were grown using a high-temperature solution growth technique^43, 44^. A mixture of La, Cr, and Ge with a molar ratio of La:Cr:Ge = 13:13:74 was premixed by arc melting. The material was then placed in a 2-mL alumina crucible and sealed in a silica ampoule under partial pressure of high purity argon gas. The sealed ampoule was heated to 1100 °C over 3 h and held for 5 h. After that, it was cooled to 825 °C and the remaining liquid was decanted using a centrifuge. Details about the crystal growth procedure and sample characterization at ambient pressure is described in ref. ^34^.

### The resistivity measurements under pressure

The samples for the pressure study were selected after ambient pressure characterization by the magnetization and resistivity measurements. Temperature and field dependent resistance measurements were carried out using a Quantum Design Physical Property Measurement System from 1.8 to 300 K. The resistivity was measured by the standard four-probe method with the current in the *ab* plane. Four Au wires with diameter of 12.5 μm were spot welded to the sample. A magnetic field, up to 9 or 14 T, was applied along the *c*-axis, which corresponds to the magnetization easy axis^34, 45^.

Two types of pressure cells were used for this experiment. A Be-Cu/Ni-Cr-Al hybrid piston-cylinder cell, similar to the one described in ref. ^46^, was used for pressures up to 2.1 GPa. A mixture of 4:6 light mineral oil: *n*-pentane^46^ used as a pressure medium, which solidifies at ~3–4 GPa at room temperature^47^. For higher pressures, a modified Bridgman cell^48^ was used to generate pressure up to 6 GPa. A 1:1 mixture of *n*-pentane:iso-pentane was used as a pressure medium. The solidification of this medium occurs around ~6–7 GPa at room temperature^47, 49^. For both cells, the pressure at low temperature was determined by the superconducting transition temperature of Pb^50^ measured by the resistivity.

The resistivity measurement under pressure at zero field is described in ref. ^16^.

### Data availability

The data that support the findings of this study are available on request from the corresponding authors.

## Electronic supplementary material

Supplementary Information
