# Supplementary material for: Tricritical wings and modulated magnetic phases in LaCrGe3 under pressure
Source: Nat Commun. 2017 Sep 15;8:546. doi: 10.1038/s41467-017-00699-x (PMC5601952; doi:10.1038/s41467-017-00699-x)
Supplement: Supplementary file 1 — Supplementary Information [file 41467_2017_699_MOESM1_ESM.pdf]

File name: Supplementary Information
Description: Supplementary Figures, Supplementary Notes and Supplementary References.

Supplementary Note 1: Metamagnetic transition from the AFM$_Q$ phase to a polarized paramagnetic state

Due to the presence of the modulated magnetic phases AFM$_Q$ or AFM$_{Q'}$, the phase diagram of $LaCrGe_3$ is complex and shows several types of metamagnetic transitions. Between 2.1 GPa and ~ 2.5 GPa and at low temperatures, the successive metamagnetic transitions are between the phases AFM$_{Q'}$, FM1, and FM2. At 2.65 GPa, AFM$_{Q'}$ and FM1 are separated by a region which is most likely a polarized paramagnetic phase (PM). The successive metamagnetic transitions are between the phases AFM$_{Q'}$, PM, FM1, and FM2. This can be seen in the field dependence of the electrical resistivity at 2.65 and 2.88 GPa shown in Supplementary Fig.1. The anomalies are clearly visible in the field derivative.

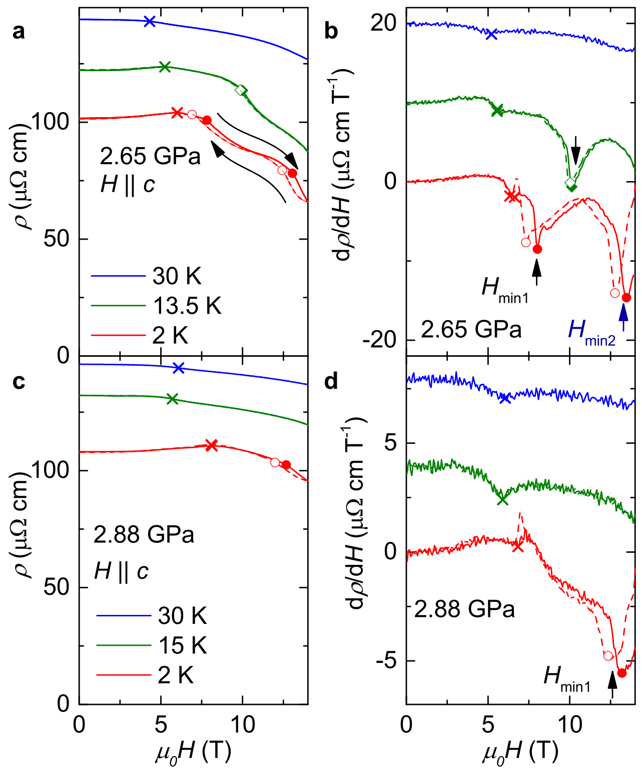

Supplementary Figure 1: **Metamagnetic transitions between AFM$_{Q'}$, PM, FM1, and FM2.** (**a**) Field dependence of the electrical resistivity at $2\,$K, $13.5\,$K, and $30\,$K at $2.65\,$GPa. (**b**) Corresponding field derivatives (dρ/dH). The curves are shifted by $10\,\mu\Omega\,$cm$\,$T^{-1} for clarity. (**c**) Field dependence of the electrical resistivity at $2\,$K, $15\,$K, and $30\,$K at $2.88\,$GPa. (**d**) Corresponding field derivatives (dρ/dH). The curves are shifted by $4\,\mu\Omega\,$cm$\,$T^{-1} for clarity. Continuous and dashed lines represent the field increasing and decreasing respectively. Vertical arrows represent the minima, which correspond to the FM1 and FM2 transitions. The cross symbols represent the transition between AFM$_{Q'}$ or AFM$_Q$ and the polarized paramagnetic phase.

Supplementary Note 2: Determination of the location of the tricritical point

In Ref. 1, the position of the tricritical point (TCP) was estimated near 40 K and 1.75 GPa based on a discontinuity in the resistivity as a function of pressure $\rho(p)$. Here, we use measurements under magnetic field to locate the TCP. When the paramagnetic-ferromagnetic (PM-FM)transition is of the second order, the magnetic field applied along the magnetization axis (c-axis) breaks the time reversal symmetry, so that no phase transition can occur. Instead, a crossover is observed resulting in a broadening and disappearing of the anomalies. Supplementary Fig. 2a, shows the peak in the temperature derivative of resistivity dρ/dT at various magnetic fields at 1.67 GPa. The peak amplitude decreases showing that the transition is of the second order. This is in contrast with the behavior at 1.83 GPa (Supplementary Fig. 2b) where the peak first increases under magnetic field indicating the first order nature of the transition. The evolution of the value of dρ/dT at the peak position as a function of magnetic field is shown in Supplementary Fig. 2c for various pressures. We can distinguish two regimes: for pressures below ~ 1.75 GPa, the peak size monotonically decreases with applied magnetic field; for pressure above 1.75 GPa, the peak size first increases with field, reach a maximum at a field H_{w} and then decreases. With this procedure, we find the TCP to be near 1.8 GPa, at which pressure the transition temperature is 40 K.

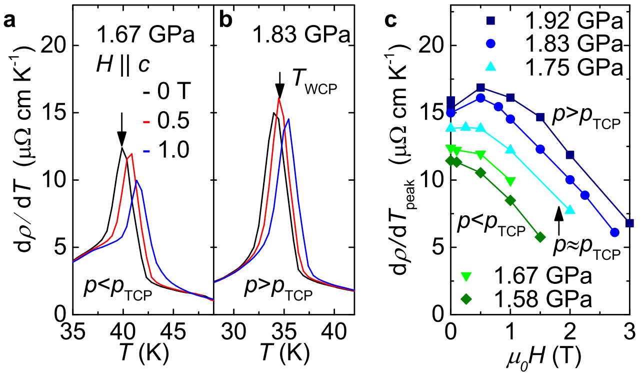

Supplementary Figure 2: **Location of the Tricritical Point.** (**a**)-(**b**) Temperature dependence of dρ/dT at various magnetic fields at 1.67 and 1.83 GPa. Arrow in panel **a** represent the T_{C} and panel **b** represent the T_{WCP}. (**c**) The variation of dρ/dT_{peak} as a function of external field for $p < p_{\mathrm{TCP}}$, $p \approx p_{\mathrm{TCP}}$ and $p_{\mathrm{TCP}} < p < p_{\mathrm{c}}$.

For $p > p_{\mathrm{TCP}}$, the location of the maximum value of dρ/dT at the peak position serves to locate the wing critical point as a function of temperature, pressure and magnetic field.

As pointed out in Supplementary Ref. 1, the fact that the TCP (near 1.8 GPa and 40 K) and the Lifshitz point (near 1.27 GPa and 56 K) are distinct points has interesting implications. Between these two points, the transition line between the FM and AFM$_Q$ phases is of the second order. This implies that the wavevector changes continuously from $Q = 0$ in the FM phase to $Q > 0$ in the AFM$_Q$ phase. In the region near this transition line, the wavevector must change its value with temperature and pressure.

Supplementary Note 3: Determination of the three-dimensional T-p-H phase diagram

In Supplementary Fig.3, we show several T-H phase diagrams at various pressures (as illustrated in Supplementary Fig. 4) . For each pressure, anomalies in the temperature and field dependence of the electrical resistivity are located and serve to outline the phase boundaries. To be complete, and for future reference, we also indicate the location of broad maxima or kinks in dρ/dT which do not seem to correspond to phase transitions at this point and are most likely related to crossover anomalies.

The T-p-H phase diagram shown as Fig.5 in the main text is constructed by combining all the T-H phase diagrams at various pressures.

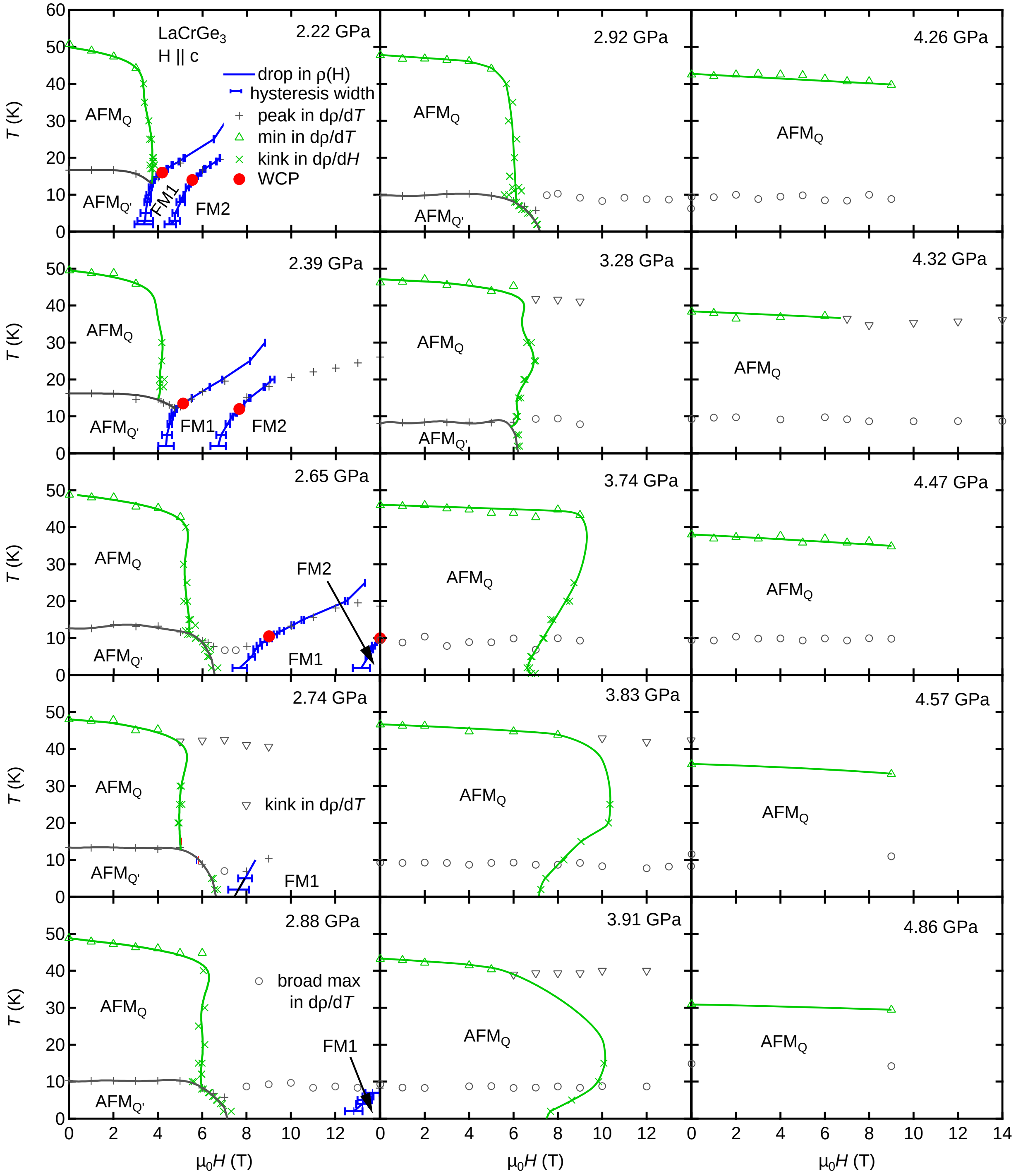

Supplementary Figure 3: T-H **phase diagrams.** Compilation of T-H phase diagrams at various pressures determined by tracking various anomalies in the temperature and field dependence of the electrical resistivity measurements up to 9 or 14 T. The hysteresis width for the drop in $\rho(H)$ (mimimum in dρ/dH) is also indicated. Lines are guides to the eyes. The pressure positions are shown in Supplementary Fig. 4.

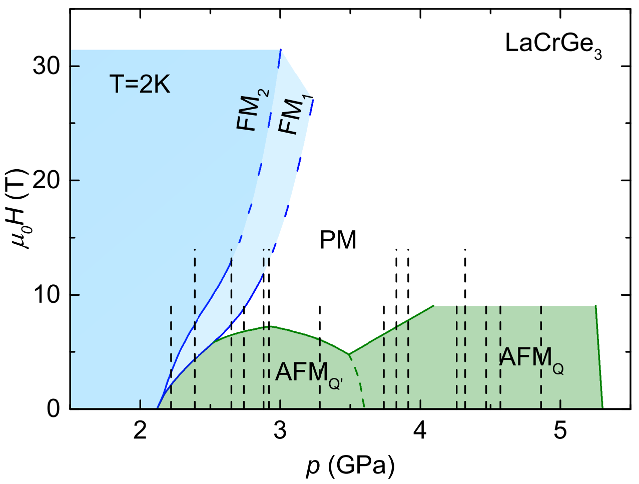

Supplementary Figure 4: p-H **phase diagram of** $\mathbf{LaCrGe_3}$ **at** 2 **K.** The black dashed lines indicate the position of the pressures for the diagrams shown in Supplementary Fig. 3. Note: Fig. 4d in the main text corresponds to the same figure limited to 3.5 GPa.

Supplementary References.

1. Taufour, V. *et al.* Ferromagnetic Quantum Critical Point Avoided by the Appearance of Another Magnetic Phase in $LaCrGe_3$ under Pressure. *Phys. Rev. Lett.* **117**, 037207 (2016).
